# Consumption and Lack of Access to Medicines and Associated Factors in the Brazilian Amazon: A Cross-Sectional Study, 2019

**DOI:** 10.3389/fphar.2020.586559

**Published:** 2020-10-06

**Authors:** Gustavo Magno Baldin Tiguman, Marcus Tolentino Silva, Taís Freire Galvão

**Affiliations:** ^1^ Faculty of Pharmaceutical Sciences, State University of Campinas, Campinas, Brazil; ^2^ Post-Graduate Program of Pharmaceutical Sciences, University of Sorocaba, Sorocaba, Brazil

**Keywords:** drug utilization, Access to Essential Medicines and Health Technologies, health services accessibility, cross-sectional studies, Brazil

## Abstract

**Objective:**

We aimed to investigate the consumption and lack of access to medicines in the adult population of Manaus, Amazonas.

**Methods:**

A population-based study was conducted in Manaus in 2019. Individuals aged ≥18 years were selected by probabilistic sampling performed in three stages. Study outcomes included the consumption of medicines in the previous fortnight and the lack of access to treatments in those who used any medicine. We calculated the prevalence ratios (PR) for the outcomes with 95% confidence intervals (CI) by Poisson regression with robust variance, considering the complex sampling design.

**Results:**

Out of the 2,321 participants, 53.2% (95%CI 50.7-55.7%) consumed medicines, of which 14.4% (95% CI 11.9–16.8%) could not obtain appropriate treatments. Analgesics were the most used medicines (557/2,702; 21.4%), whereas antibiotics were the most inaccessible treatments (18/228; 7.9%). Lack of financial resources was the main reason for not accessing treatments (104/228; 45.6%). Consumption was significantly associated with older age (≥60 years: PR = 1.27; 95%CI 1.09–1.49), lower social class (D/E: PR = 0.84; 95%CI 0.72–0.99), lower educational level (p = 0.039), poor health status (PR = 1.30; 95%CI 1.11–1.52), use of health care services (PR = 1.37; 95%CI 1.26–1.49), and chronic diseases (PR = 1.36; 95%CI 1.22–1.52). Lack of access was higher in people with poor health status (PR = 2.46; 95%CI 1.50–4.04) and chronic diseases (PR = 1.84; 95%CI 1.16–2.92).

**Conclusion:**

Half of Manaus’ population used medicines, which was higher in socially privileged and sicker individuals. Among those, 14 in every 100 could not access drug therapies, which was more frequent in people with poor health and with chronic diseases.

## Introduction

The use and access to medicines are important indicators of population health due to their role in promoting health maintenance and reducing global morbidity and mortality (Ofori-Asenso, 2016). Non-communicable chronic diseases and multimorbidity are increasingly prevalent globally (James et al., 2018), which is particularly alarming in vulnerable settings (Araujo et al., 2018). Expansion of the access to medicines and innovations on pharmacological treatments are powerful strategies to decrease the burden of chronic diseases (Mokdad et al., 2018; Lichtenberg, 2019).

Nearly 2 billion people have no access to basic medicines, which results in greater suffering, prolonged illness, needless disabilities, and preventable deaths (Chan, 2017). Access to high-quality essential medicines with affordable prices is essential to reduce the financial burden of care and improve the population health worldwide (Ozawa et al., 2019).

In health systems, access to medicines depends on five main dimensions: availability, affordability, accessibility, acceptability, and quality (Bigdeli et al., 2013; Wirtz et al., 2016). The Unified Health System (*Sistema Único de Saúde*, SUS), the Brazilian public health care system, aims to provide free and universal access to health care services, equity of care, and integrality of health assistance to all citizens (Viacava et al., 2018). Medicines represent important health expenditures in Brazilian households, especially for socioeconomically disadvantaged families (Luiza et al., 2016). The free availability of medicines and access to health care services from SUS are imperative for these individuals (Andrade et al., 2018), but previous studies indicate that the Brazilian pharmaceutical policy is not ensuring access to essential medicines to the entire population (Bertoldi et al., 2012). Even if prescribed, essential medicines may not be available at SUS due to deficiencies in the governmental supply chain management, including constraints in the funding of pharmaceutical policies (Nascimento et al., 2017a).

Brazil lacks information systems that can provide data about the utilization of medicines by the general population. Population-based cross-sectional studies can be useful tools to investigate the consumption of medicines and the lack of access to treatments in relation to the population’s sociodemographic and clinical characteristics, in particular for underserved regions, such as the Brazilian Amazon. The aim of this study was to investigate the prevalence of consumption and lack of access to medicines among adults in the Brazilian Amazon.

## Materials and Methods

### Study Design

A population-based cross-sectional study was conducted between April and June 2019 with adults living in Manaus, Amazonas. This study is part of a major survey that aimed to investigate the use of health care services and supplies in the city (Silva et al., 2019).

### Setting

Manaus is the capital of Amazonas state, located in the North region of Brazil. According to the national estimates, Manaus had 2,106,322 inhabitants in 2018, which corresponded to more than 50% of Amazonas’ population (IBGE, 2018). Manaus was in the 850^th^ position on the Human Development Index among 5,570 Brazilian cities in 2010 (Atlas of Human Development, 2013) and in the 8^th^ Brazilian city position for Gross Domestic Product in 2016 (IBGE, 2018).

### Participants and Sample Size

The participants were selected through a probabilistic sampling method performed in three stages, stratified by sex and age: census tracts (random), household (systematic), and individual (random) (Silva et al., 2019). The sample size was calculated considering a prevalence of health care services usage in the past 15 days of 20% in the region (Silva and Galvao, 2017), a confidence level of 95%, an absolute precision of 2%, and the estimates of 2,106,322 adult residents (IBGE, 2018). In total, 2,300 individuals were planned to participate in the study (Silva et al., 2019).

### Variables

The primary outcome was the consumption of medicines in the previous 15 days. The secondary outcome was the lack of access to treatments, defined as the unmet need for pharmacological treatments among those who reported using medicines in the period. Independent variables included: sex (male, female), age group (18–24, 25–34, 34–44, 45–59, and ≥60 years old), ethnicity (white/Asian, non-white/Asian), economic classification (A/B, C, D/E, where A refers to the wealthiest and E to the poorest according to the Brazilian Economic Criteria (ABEP, 2018)), educational level (higher education or above, high school, elementary school, less than elementary school), self-perception of health status (good, fair, poor), health insurance (no, yes), seek for a health care services in the previous 15 days (no, yes, considering both public and private services), and presence of chronic diseases (no, yes, including hypertension, diabetes, hypercholesterolemia, heart diseases, stroke, asthma, arthritis, chronic back pain, depression, mental illnesses, lung diseases, cancer, and chronic renal failure).

### Data Sources and Measurement

The consumption of medicines in the previous 15 days was assessed by the following question: “In the last 15 days (or two weeks), did you take any medicine?”. In case of a positive answer, the name of the medicine, the condition/disease, the duration of treatment, the person who indicated the medicine, and the method for obtaining the medicine were recorded. Among individuals who reported consumption of medicines, the lack of access to treatments was measured by the question: “Is there any medicine that you should be taking, but you are not?”. If the answer was positive, the name of the medicine, the condition/disease, the person who recommended or prescribed the medicine, and the reasons for not using it were recorded. After data collection, the medicines were classified in accordance with the Anatomical Therapeutic Chemical (ATC) Classification System of the World Health Organization (WHO, 2019). All medicines were classified in their full ATC code (all levels). In cases of indecipherable writing or unavailability of the product at the ATC, we classified the medicines as “non-codifiable”. The name of the medicine was optionally confirmed in the medical prescriptions or drug packages if available at the moment of the interview.

A team of interviewers with experience in quantitative studies was hired and trained by the project researchers, who collected data through face-to-face interviews at the participant’s household. Questionnaires were pre-configured in the software SurveyToGo (Dooblo Ltd, Israel) and data were recorded using electronic devices (Intel TabPhone 710 Pro). The participants’ responses were transmitted to a study database *via* internet.

### Bias

We considered the consumption of medicines in the last 15 days prior to the interview to avoid recall bias. If available at the households, the consumed medicines were confirmed by the medical prescription or drug package. We conducted a pilot study with 150 participants to evaluate their understanding of the questionnaire, who were included in the final sample. To assess the validity of the collected data, 20% of the interviews were audited by phone. The audio of the interviews was recorded and the location where the interviews were conducted was georeferenced by the device.

### Statistical Analysis

We used descriptive statistics to characterize the sample. The pharmacological or therapeutic subgroups (second level of the ATC) were described according to their frequency. The prevalence ratios (PR) of medicine consumption and lack of access to treatments by independent variables were calculated using Poisson regression with robust variance with 95% confidence intervals (CI). Variables with p-value<0.20 in the crude analysis were included in the adjusted multivariate regression. Wald test was used to assess the significance of the variables in multiple categories; variables with p-value<0.05 were considered statistically significant. All analyses were conducted in Stata 14.2 software, taking into account the complex sampling design (svy command).

### Ethics

This study was approved by the Ethics Research Committee from the University of Amazonas through the approval letter No. 3.102.942 from 28 December, 2018 (Certificate of Presentation for Ethical Appreciation at Brazil Platform: 04728918.0.0000.5020). All of the participants signed an Informed Consent Form before any study procedure was performed.

## Results

Out of the 2,321 participants who were included in the study (**Supplementary Material 1**), 1,276 used medicines in the previous 15 days (prevalence: 53.2%; 95% CI 50.7–55.7%), of which 176 reported lack of access to treatments (prevalence: 14.4%; 95% CI 11.9–16.8%).

Just over half of the participants were men (51.0%), aged 18 to 34 years (52.2%), and belonged to middle-class (53.7%). Most of the individuals were non-White/Asian (85.0%), completed at least high school (59.4%), self-reported their health statuses as good (67.2%), had no health insurance (85.5%), did not use health care services in the previous 15 days (71.4%), and had chronic diseases (57.1%; **Table 1**).

**Table 1:** Main characteristics of the participants and consumption and lack of access to medicines among the adult population of Manaus in 2019 (N=2,321), adjusted for the complex sampling design.

Variables		Population		Consumption of medicines^a^		Lack of access to medicines^a^ ^, ^ ^b^	
		n	%	n	%	n	%
Sex							
	Men	1,088	51.0	570	48.6	54	36.4
	Women	1,233	49.0	706	51.4	122	63.6
Age group (years)							
	18–24	405	20.3	201	17.7	29	18.1
	25–34	586	31.9	303	30.9	33	25.4
	35–44	553	22.2	271	20.3	34	22.6
	45–59	526	18.9	322	21.9	50	22.7
	≥ 60	251	6.7	179	9.2	30	11.2
Ethnicity							
	White/Asian	349	15.0	192	15.3	19	15.4
	Non-White/Asian	1,972	85.0	1,084	84.7	157	84.6
Economic classification^c^							
	A/B	282	13.4	171	15.0	17	11.1
	C	1,244	53.7	689	54.6	88	47.6
	D/E	795	32.9	416	30.4	71	41.3
Educational level							
	Higher education or above	153	6.9	103	8.6	8	5.1
	High school	1,171	52.5	627	52.0	80	44.7
	Elementary school	432	20.4	226	18.6	33	19.5
	Less than elementary school	565	20.2	320	20.8	55	30.7
Health status							
	Good	1,498	67.2	709	57.8	63	34.3
	Fair	671	26.8	457	34.0	82	47.2
	Poor	152	6.0	110	8.2	31	18.5
Health Insurance							
	No	1,978	85.5	1,072	84.1	151	88.2
	Yes	343	14.5	204	15.9	25	11.8
Seek for healthcare services^a^							
	No	1,623	71.4	772	62.2	88	51.4
	Yes	698	28.6	504	37.8	88	48.6
Chronic diseases							
	No	921	42.9	374	32.5	27	15.6
	Yes	1,400	57.1	902	67.5	149	84.4
Total		2,321	100.0	1,276	100.0	176	100.0

a In the previous 15 days.

b Among those who consumed medicines in the previous 15 days (N=1,276).

c According to the Brazilian Economic Criteria, where A refers to the wealthiest and E to the poorest.

The consumption of medicines was more frequent among women (51.4%), individuals aged 25 to 34 years (30.9%), non-white/Asian people (84.7%), those belonging to middle-class (54.6%), individuals who completed high school (60.6%), people with good health status (57.8%) and no health insurance (84.1%), those who did not use health care services in the previous 15 days (62.2%), and people with chronic diseases (67.5%). Lack of access to treatments was higher in women (63.6%), non-White/Asian individuals (84.6%), poor people (88.9%), individuals with complete high school (44.7%), those with fair health status (47.2%), people with no health insurance (88.2%), and individuals with chronic diseases (84.4%).

Analgesics (577/2,702; 21.4%) were the most used therapeutic class of medicines, followed by anti-inflammatory and antirheumatic drugs (247/2,702; 9.1%) and antibiotics (227/2,702; 8.4%). Antibiotics (18/228; 7.9%), vitamins (15/228; 6.6%), and anti-inflammatory and antirheumatic medicines (13/228; 5.7%) were the most inaccessible treatments (**Table 2**).

**Table 2:** Description of most consumed medicines (N=2,702 medicines) and most inaccessible treatments (N=228 medicines) in the 15 days prior to interview in Manaus, 2019.

Medicine	ATC Code^a^	Consumption of medicines (N=2,702)		Lack of access to medicines (N=228)	
		n	%	n	%
Analgesics	N02	577	21.4	8	3.5
Anti-inflammatory and antirheumatic products	M01	247	9.1	13	5.7
Antibacterials for systematic use	J01	227	8.4	18	7.9
Agents acting on the renin-angiotensin system	C09	216	8.0	4	1.8
Drugs used in diabetes	A10	142	5.3	10	4.4
Muscle relaxants	M03	119	4.4	–	–
Drugs for acid-related disorders	A02	92	3.4	8	3.5
Vitamins	A11	71	2.6	15	6.6
Lipid modifying agents	C10	69	2.6	12	5.3
Anti-anemic preparations	B03	60	2.2	12	5.3
Sex hormones and modulators of the genital system	G03	49	1.8	7	3.1
Antihypertensives	C02	27	1.0	4	1.8
Anti-epileptics	N03	25	0.9	5	2.2
Other	–	781	28.9	112	49.1

ATC, Anatomical Therapeutic Chemical Classification System.

a According to the 2^nd^ level (Pharmacological or Therapeutic subgroup) of the ATC.

Out of the 2,702 medicines used among the participants, 68.1% (1,839/2,702) were recommended or prescribed by a physician, 19.1% (515/2,702) were used based on self-knowledge, and 6.9% (187/2,702) were recommended by a pharmacist or a pharmacy clerk (**Supplementary Material 2**).

The main reported reasons for not accessing treatments were lack of financial resources (104/228; 45.6%), unavailability of medicines in the health care services or pharmacy (36/228; 15.8%), and lack of medical prescription [15/228; 6.6% (**Table 3** **)**].

**Table 3:** Reasons for the lack of access to medicines (N=228) in the 15 days prior to the interview in Manaus, 2019.

Main reasons	N	%
Lack of financial resources	104	45.6
Medicine unavailable in the healthcare service or pharmacy	36	15.8
Lack of medical prescription	15	6.6
Adverse events	14	6.1
Unwillingness to take the medicine	12	5.3
Forgot to obtain the medicine	10	4.4
Other	37	16.2

After adjustment, the consumption of medicines in the previous 15 days was positively associated with older age (≥60 years: PR 1.27; 95% CI 1.09–1.49), fair (PR 1.27; 95% CI 1.16–1.40) and poor (PR 1.30; 95% CI 1.11–1.52) health statuses, use of health care services in the previous 15 days (PR 1.37; 95% CI 1.26–1.49), and presence of chronic diseases (PR 1.36; 95% CI 1.22–1.52), while negatively associated with lower social class (D/E: PR 0.84; 95% CI 0.72–0.99) and lower educational level (high school: PR 0.85, 95% CI 0.72–0.99; elementary school: PR 0.78, 95% CI 0.65–0.94; less than elementary school: PR 0.78, 95% CI 0.65–0.94). The lack of access to treatments was positively associated with fair (PR 1.82; 95% CI 1.23–2.69) and poor (PR 2.46; 95% CI 1.50–4.04) health statuses, and presence of chronic diseases (PR 1.84; 95% CI 1.16–2.92; **Table 4**).

**Table 4:** Unadjusted and adjusted prevalence ratios (PRs) and 95% confidence intervals (CIs) of consumption and lack of access to medicines in Manaus, 2019 (N=2,321).

Variables		Consumption of medicines^a^				Lack of access to medicines^a^			
		PR (95% CI)	p-value	Adjusted PR (95% CI)	p-value	PR (95% CI)	p-value	Adjusted PR (95% CI)	p-value
Sex			0.048		0.902		0.007		0.050
	Men	1.00		1.00		1.00		1.00	
	Women	1.10 (1.00–1.21)		1.01 (0.92–1.10)		1.65 (1.14–2.39)		1.43 (1.00–2.04)	
Age group (years)			<0.001		<0.001		0.714		-^b^
	18–24	1.00		1.00		1.00		-^b^	
	25–34	1.11 (0.95–1.29)		1.04 (0.89–1.21)		0.80 (0.47–1.37)		-^b^	
	35–44	1.05 (0.89–1.23)		0.92 (0.79–1.08)		1.09 (0.64–1.86)		-^b^	
	45–59	1.33 (1.15–1.53)		1.08 (0.93–1.25)		1.01 (0.62–1.65)		-^b^	
	≥ 60	1.58 (1.36–1.83)		1.27 (1.09–1.49)		1.18 (0.67–2.10)		-^b^	
Ethnicity			0.724		-^b^		0.976		-^b^
	White/Asian	1.00		-^b^		1.00		-^b^	
	Non-White/Asian	0.98 (0.86–1.11)		-^b^		0.99 (0.57–1.72)		-^b^	
Economic classification^c^			0.032		0.083		0.020		0.404
	A/B	1.00		1.00		1.00		1.00	
	C	0.91 (0.79–1.04)		0.92 (0.80–1.06)		1.18 (0.67–2.07)		1.00 (0.56–1.78)	
	D/E	0.83 (0.71–0.96)		0.84 (0.72–0.99)		1.84 (1.04–3.24)		1.26 (0.68–2.35)	
Educational level			0.004		0.039		0.032		0.822
	Higher education or above	1.00		1.00		1.00		1.00	
	High school	0.79 (0.68–0.92)		0.85 (0.72–0.99)		1.46 (0.57–3.72)		1.33 (0.55–3.22)	
	Elementary school	0.73 (0.61–0.87)		0.78 (0.65–0.94)		1.78 (0.67–4.73)		1.51 (0.58–3.95)	
	Less than elementary school	0.82 (0.70–0.97)		0.78 (0.65–0.94)		2.49 (0.96–6.48)		1.50 (0.58–3.89)	
Health status			<0.001		<0.001		<0.001		<0.001
	Good	1.00		1.00		1.00		1.00	
	Fair	1.47 (1.34–1.61)		1.27 (1.16–1.40)		2.34 (1.62–3.38)		1.82 (1.23–2.69)	
	Poor	1.58 (1.38–1.81)		1.30 (1.11–1.52)		3.83 (2.46–5.96)		2.46 (1.50–4.04)	
Health insurance			0.091		0.371		0.132		0.609
	No	1.00		1.00		1.00		1.00	
	Yes	1.11 (0.98–1.26)		1.06 (0.94–1.19)		0.71 (0.45–1.11)		0.89 (0.57–1.40)	
Seek for healthcare services^a^			<0.001		<0.001		0.010		0.199
	No	1.00		1.00		1.00		1.00	
	Yes	1.52 (1.40–1.66)		1.37 (1.26–1.49)		1.55 (1.11–2.17)		1.24 (0.89–1.72)	
Chronic diseases			<0.001		<0.001		<0.001		0.010
	No	1.00		1.00		1.00		1.00	
	Yes	1.56 (1.40–1.73)		1.36 (1.22–1.52)		2.61 (1.66–4.10)		1.84 (1.16–2.92)	

a In the previous 15 days.

b Removed from the multivariate regression (p>0.20 in unadjusted analysis).

c According to the Brazilian Economic Criteria, where A refers to the wealthiest and E to the poorest.

## Discussion

Half of the adult population of Manaus used medicines in the previous 15 days, which was associated with older age, lower social class and educational level, worse health statuses, use of health care services, and presence of chronic diseases. Among those who used medicines, 14 in every 100 reported lack of access to treatments; unmet need for medicines was higher people with worse health statuses and people with chronic diseases.

This study presents limitations, such as those present in cross-sectional designs (Thiese, 2014). Recall bias may have underestimated the results as the participants may have forgotten to report the use of some medicines. Since the study was conducted in the first semester, seasonality may have also influenced the results as some diseases (e.g. bacterial and viral infections) have higher incidences in the rainy season, especially in areas of high humidity (Choe et al., 2019; Price et al., 2019). In the present study, drugs were not restricted only to essential medicines, thus policy makers should interpret our results with caution. Sample size calculations were performed considering estimates of health care services usage; therefore, the survey was not specifically powered based on the outcomes of the present analysis.

The prevalence of medicine consumption found in our study was similar to the one reported in a systematic review conducted in Brazil, about 50% (Gomes et al., 2017). Our study found an increased lack of access to treatments when compared to a population-based study conducted in Porto Alegre, Southern Brazil with 2,988 individuals in 2003, which found that 5% of the participants who used medicines in the 15 days prior to the interview could not obtain appropriate treatment (Bertoldi et al., 2009). Previous data from population-based studies reinforce the differences in the access to treatments between Brazilian regions, particularly in the North and Northeast regions, the least developed of the country (Paniz et al., 2008; Goes et al., 2016; Alvares et al., 2017).

We found that the use of medicines was associated with older age, similarly to the results of a population-based cross-sectional study conducted with 1,720 adults from Florianópolis, Southern Brazil in 2009, which suggested that the prevalence of medicine use was higher in older individuals (Boing et al., 2011). The promotion of rational use of medicines among the elderly is particularly important since older age is associated with higher rates of potentially inappropriate medications (Lutz et al., 2017), as well as polypharmacy and self-medication (Santos et al., 2013; Ramos et al., 2016; Nascimento et al., 2017b).

Consumption of medicines was negatively associated with lower social class, similar to a previous national survey with 41,433 people, which observed that the lowest prevalence of medicine consumption occurred in poorer individuals and in the North region of Brazil – where Manaus is located (Bertoldi et al., 2016). Indeed, previous analyses conducted in this region showed that socioeconomic inequalities play an important role in the access and use of health care services among adults (Araujo et al., 2017; Galvao et al., 2019). In a previous 2015 survey of 4,001 adults from Manaus and surrounding cities, the prevalence of medicine consumption was 29%, which was not associated with higher social status as observed in the present research (Lima et al.). Since then, dismantling of public policies related to the access to essential medicines (Trindade, 2018) may explain the current influence of socioeconomic variables in the use of medicines in present analysis. The lower use of medicines among the poor may have worsened due to the recent changes in the *Farmácia Popular* program – a governmental initiative created in 2004 with the aim of increasing access to medicines with lower prices –, which could potentially result in higher expenditures with treatments for several Brazilian families (Trindade, 2018). In the present study, as consumption was not restricted only to essential medicines provided by SUS, it is possible that individuals from higher social classes used more medicines due to their higher purchasing power, as indicated by a previous analysis (Vogler et al., 2015).

We also found that the use of medicines was lower among those with lower educational level. This contrasts with the results of a national cross-sectional study conducted with 8,803 patients from the Brazilian Unified Health System in 2015, which indicated that the majority of those who consumed medicines in the previous 30 days had lower educational levels (Costa et al., 2017). The results from the Brazilian National Health Survey from 2013 also endorses our findings: the percentage of individuals who obtained all medications prescribed in the last health care visit was higher among people with higher levels of education in comparison to those with lower educational attainment (Stopa et al., 2017).

The use of medicines was higher in people with worse health statuses, chronic diseases, and who used health care services recently. A national cross-sectional study conducted in an adult sample in Spain also found that medication use was significantly associated with negative perception of health status, presence of chronic diseases, and visits to the physician (Martín-Pérez et al., 2015).

Nearly one-fourth of the medicines were used by self-medication or indication by a family member/neighbor, which is higher than the prevalence found in a previous national cross-sectional study of 16.1% (Arrais et al., 2016). These findings may be explained by the significant inequities in the access to health care services (Araujo et al., 2017; Galvao et al., 2019) and the long waiting times for medical consultations in the region (Galvao et al., 2020), as high health care costs and long waiting times can be predictors of self-medication behavior (Shaghaghi et al., 2014).

Lack of financial resources was the most reported reason for not accessing treatments in our study. A population-based study conducted in Brazil in 2009 found similar results: in 22% of the households, medicines were not acquired due to lack of financial resources, which was higher in the North and Northeast regions (Goes et al., 2016). The high prices of medicines can increase catastrophic expenditures for patients, especially in low- and middle-income countries (Ahmadiani and Nikfar, 2016). In the present analysis, the unavailability of medicines in health care services and pharmacies was also a common reason for the lack of access. Previous analyses showed that the availability of medicines, notably in SUS, does not supply essential medicines to the entire population, which impacts mainly the poorest people (Bertoldi et al., 2012).

Our study also showed that individuals with worse health statuses and with chronic diseases had lower access to required treatment, that is, the sicker individuals were not accessing all the medicines they needed. In a national population-based study conducted in 2014 with 12,725 people who reported having chronic diseases, lack of access was highest in the North region, and people with worse health status had significantly less access to treatment (Oliveira et al., 2016). The South and Southeast regions of Brazil also have greater access to health care services, especially when compared to underserved areas, such as the Amazon (Stopa et al., 2017). The sicker inhabitants of Manaus have lower access to health care services and appropriate treatment, which may result in higher expenditures from both individual and health system perspectives (Luiza et al., 2016; Massuda et al., 2018).

Participants mostly used symptomatic drugs, antibiotics, and drugs for the treatment of hypertension and diabetes in the two weeks prior the interview. Some of these therapeutic classes were also frequently not accessible. Although we could not assess if the drug therapies were appropriate, it is possible that the lack or insufficiency of treatment of previously minor conditions could increase the burden of diseases and related costs (Fielding et al., 2015). Lack of access was measured only in individuals who reported using at least one medicine in the recall period to avoid memory bias; therefore, lack of access to medicines is potentially underestimated in our analysis. The restriction to people using medicines recently may reflect decisions that this sample had to make when obtaining the products since the main reason for not assessing treatments was the lack of money, such as the prioritization to buy cheaper medicines (Weiner, 2001; Gronde et al., 2017) and the request of advice from community pharmacists to avoid purchasing ineffective or potentially harmful treatments (Dalton and Byrne, 2017).

Antibiotics were reported as the main inaccessible treatments. Brazilian federal expenditures on anti-infectives for systemic use and drugs for alimentary tract and metabolism significantly decreased from 2007 to 2014, whereas expenditures on immunomodulating agents increased (Magarinos-Torres et al., 2017). While the allocation of resources on specific therapeutic areas indicates governmental and industrial health priorities, it may also restrict the population’s access to certain medications (Stolk et al., 2006; Brall and Schroder-Back, 2016). Interestingly, an analysis conducted in Manaus showed that self-medication with antibiotics increased between 2015 and 2019, suggesting that the population had to seek for alternative sources to obtain treatment and that self-medication persists even after the regulation of antibiotics sales was implemented (Baldin Tiguman et al., 2020).

In conclusion, half of the adult population of Manaus used medicines in the previous 15 days, which was higher in socially privileged people and sicker individuals. Lack of access to treatments was reported by 14 out of 100 inhabitants who consumed medicines and was higher in people with worse health statuses, and people with chronic diseases. Higher investments in pharmaceutical services and access policies are necessary to improve the rational consumption and proper access to medicines in the region.

## Data Availability Statement

The raw data supporting the conclusions of this article will be made available by the authors, without undue reservation.

## Ethics Statement

The studies involving human participants were reviewed and approved by Ethics Research Committee from the University of Amazonas. The patients/participants provided their written informed consent to participate in this study.

## Author Contributions

MS and TG designed the work, analyzed and interpreted the data, critically reviewed the work for important intellectual content, and approved the final version of this manuscript. GT analyzed and interpreted the data and drafted the work. All authors contributed to the article and approved the submitted version.

## Funding

This research was funded by the Brazilian Council for Scientific and Technological Development – CNPq (Grants No. 404990/2013-4 and 448093/2014-6). Galvao TF receives productivity scholarship from CNPq (Grant No. 3064482017-7).

## Conflict of Interest

The authors declare that the research was conducted in the absence of any commercial or financial relationships that could be construed as a potential conflict of interest.

## References

[B1] ABEP (2018). Critérios Brasileiros de Classificação Econômica 2018 (Associação Brasileira de Empresas de Pesquisa). Available at: http://www.abep.org/criterio-brasil (Accessed 20 Setembro 2019 2019).

[B2] Ahmadiani S. Nikfar S. (2016). Challenges of access to medicine and the responsibility of pharmaceutical companies: a legal perspective. Daru 24 (1), 13. 10.1186/s40199-016-0151-z 27141958 PMC4855755

[B3] Alvares J. Guerra A. A. J. Araujo V. E. Almeida A. M. Dias C. Z. Ascef B. O. (2017). Access to medicines by patients of the primary health care in the Brazilian Unified Health System. Rev. Saude Publica 51 (suppl 2), 20s. 10.11606/S1518-8787.2017051007139 29160463 PMC5676371

[B4] Andrade M. V. Coelho A. Q. Xavier Neto M. Carvalho L. R. Atun R. Castro M. C. (2018). Brazil’s Family Health Strategy: factors associated with programme uptake and coverage expansion over 15 years, (1998-2012). Health Policy Plan 33 (3), 368–380. 10.1093/heapol/czx189 29346551

[B5] Araujo M. E. A. Silva M. T. Galvao T. F. Pereira M. G. (2017). Prevalence of health services usage and associated factors in the Amazon region of Brazil: a population-based cross-sectional study. BMJ Open 7 (11), e017966. 10.1136/bmjopen-2017-017966 PMC5701988 29151052

[B6] Araujo M. E. A. Silva M. T. Galvao T. F. Nunes B. P. Pereira M. G. (2018). Prevalence and patterns of multimorbidity in Amazon Region of Brazil and associated determinants: a cross-sectional study. BMJ Open 8 (11), e023398. 10.1136/bmjopen-2018-023398 PMC6231594 30391918

[B7] Arrais P. S. Fernandes M. E. Pizzol T. D. Ramos L. R. Mengue S. S. Luiza V. L. (2016). Prevalence of self-medication in Brazil and associated factors. Rev. Saude Publica 50 (suppl 2), 13s. 10.1590/S1518-8787.2016050006117 PMC5157904 27982373

[B8] Atlas of Human Development (2013). Atlas of Human Development in Brazil. Manaus, AM (United Nations Development Programme: Institute of Applied Economic Research. João Pinheiro Foundation). Available at: http://www.atlasbrasil.org.br/2013/pt/perfil_m/manaus_am (Accessed 6 November 2019 2019).

[B9] Baldin Tiguman G. M. Silva M. T. Galvao T. F. (2020). Use and self-medication with antibiotics among adults in the Brazilian Amazon: a panel of two cross-sectional studies 2015 and 2019. Expert Rev. Anti-Infective Ther. 10.1080/14787210.2020.1798228 32700582

[B10] Bertoldi A. D. de Barros A. J. Wagner A. Ross-Degnan D. Hallal P. C. (2009). Medicine access and utilization in a population covered by primary health care in Brazil. Health Policy 89 (3), 295–302. 10.1016/j.healthpol.2008.07.001 18722031

[B11] Bertoldi A. D. Helfer A. P. Camargo A. L. Tavares N. U. Kanavos P. (2012). Is the Brazilian pharmaceutical policy ensuring population access to essential medicines? Global Health 8:6. 10.1186/1744-8603-8-6 22436555 PMC3511298

[B12] Bertoldi A. D. Pizzol T. D. Ramos L. R. Mengue S. S. Luiza V. L. Tavares N. U. (2016). Sociodemographic profile of medicines users in Brazil: results from the 2014 PNAUM survey. Rev. Saude Publica 50 (suppl 2), 5s. 10.1590/S1518-8787.2016050006119 PMC5157907 27982375

[B13] Bigdeli M. Jacobs B. Tomson G. Laing R. Ghaffar A. Dujardin B. (2013). Access to medicines from a health system perspective. Health Policy Plan 28 (7), 692–704. 10.1093/heapol/czs108 23174879 PMC3794462

[B14] Boing A. C. Bertoldi A. D. Peres K. G. (2011). Socioeconomic inequalities in expenditures and income committed to the purchase of medicines in Southern Brazil. Rev. Saude Publica 45 (5), 897–905. 10.1590/s0034-89102011005000054 21829974

[B15] Brall C. Schroder-Back P. (2016). Personalised medicine and scarce resources: a discussion of ethical chances and challenges from the perspective of the capability approach. Public Health Genomics 19 (3), 178–186. 10.1159/000446536 27238357 PMC5296898

[B16] Chan M. (2017). en Years in Public Health, 2007-2017— Access to Medicines: Making Market Forces Serve the Poor (Geneva, Switzerland: World Health Organization). Available at: https://www.who.int/publications/10-year-review/chapter-medicines.pdf?ua=1 (Accessed 21 May 2020 2020).

[B17] Choe Y. J. Smit M. A. Mermel L. A. (2019). Seasonality of respiratory viruses and bacterial pathogens. Antimicrob. Resist. Infect. Control 8, 125. 10.1186/s13756-019-0574-7 31367346 PMC6647268

[B18] Costa C. M. F. N. Silveira M. R. Acurcio F. D. A. Guerra Junior A. A. Guibu I. A. Costa K. S. (2017). Use of medicines by patients of the primary health care of the Brazilian Unified Health System. Rev. Saúde Pública 51 (suppl 2), 18s. 10.11606/s1518-8787.2017051007144 29160464 PMC5676385

[B19] Dalton K. Byrne S. (2017). Role of the pharmacist in reducing healthcare costs: current insights. Integr. Pharm. Res. Pract. 6, 37–46. 10.2147/IPRP.S108047 29354549 PMC5774321

[B20] Fielding S. Porteous T. Ferguson J. Maskrey V. Blyth A. Paudyal V. (2015). Estimating the burden of minor ailment consultations in general practices and emergency departments through retrospective review of routine data in North East Scotland. Fam. Pract. 32 (2), 165–172. 10.1093/fampra/cmv003 25742695 PMC4371893

[B21] Galvao T. F. Tiguman G. M. B. Caicedo Roa M. Silva M. T. (2019). Inequity in utilizing health services in the Brazilian Amazon: A population-based survey 2015. Int. J. Health Plann. Manage. 10.1002/hpm.2902 31515900

[B22] Galvao T. F. Tiguman G. M. B. Costa Filho D. B. D. Silva M. T. (2020). Waiting time and medical consultation length in the Manaus metropolitan region, Brazil: a cross-sectional, population-based study 2015. Epidemiol. Serv. Saude 29 (4), e2020026. 10.5123/s1679-49742020000400014 32785433

[B23] Goes F. C. Homem-de-Mello M. Caldas E. D. (2016). Access to medicines in Brazil based on monetary and non-monetary acquisition data obtained from the 2008/2009 Household Budget Survey. Rev. Saude Publica 50, 79. 10.1590/S1518-8787.2016050006635 28099666 PMC5167100

[B24] Gomes V. P. Silva M. T. Galvao T. F. (2017). Prevalence of medicine use among Brazilian adults: a systematic review. Cien Saude Colet 22 (8), 2615–2626. 10.1590/1413-81232017228.29412016 28793077

[B25] Gronde T. V. Uyl-de Groot C. A. Pieters T. (2017). Addressing the challenge of high-priced prescription drugs in the era of precision medicine: a systematic review of drug life cycles, therapeutic drug markets and regulatory frameworks. PloS One 12 (8), e0182613. 10.1371/journal.pone.0182613 28813502 PMC5559086

[B26] IBGE (2018). Manaus (Instituto Brasileiro de Geografia e Estatística). Available at: https://cidades.ibge.gov.br/brasil/am/manaus/panorama (Accessed 20 Setembro 2019 2019).

[B27] James S. L. Abate D. Abate K. H. Abay S. M. Abbafati C. Abbasi N. (2018). Global, regional, and national incidence, prevalence, and years lived with disability for 354 diseases and injuries for 195 countries and territories 1990–2017: a systematic analysis for the Global Burden of Disease Study 2017. Lancet 392 (10159), 1789–1858. 10.1016/s0140-6736(18)32279-7 30496104 PMC6227754

[B28] Lichtenberg F. R. (2019). The impact of pharmaceutical innovation on the burden of disease in Canada 2000-2016. SSM Popul. Health 8, 100457. 10.1016/j.ssmph.2019.100457 31440578 PMC6698939

[B29] Lima V. G. Silva M. T. Galvao T. F. Medication use by adults in greater Manaus: a population-based cross-sectional study 2015 (in press). Braz. J. Pharm. Sci.

[B30] Luiza V. L. Tavares N. U. L. Oliveira M. A. Arrais P. S. D. Ramos L. R. Pizzol T. D. S. D. (2016). Catastrophic expenditure on medicines in Brazil. Rev. Saúde Pública 50 (suppl 2), 15s. 10.1590/s1518-8787.2016050006172 PMC5157912 27982383

[B31] Lutz B. H. Miranda V. I. A. Bertoldi A. D. (2017). Potentially inappropriate medications among older adults in Pelotas, Southern Brazil. Rev. Saúde Pública 51, 52. 10.1590/s1518-8787.2017051006556 28658367 PMC5493363

[B32] Magarinos-Torres R. Lynd L. D. Luz T. C. B. Marques P. Osorio-de-Castro C. G. S. (2017). Essential Medicines List Implementation Dynamics: A Case Study Using Brazilian Federal Medicines Expenditures. Basic Clin. Pharmacol. Toxicol. 121 (3), 181–188. 10.1111/bcpt.12783 28371342

[B33] Martín-Pérez M. Hernández Barrera V. López de Andrés A. Jiménez-Trujillo I. Jiménez-García R. Carrasco-Garrido P. (2015). Predictors of medication use in the Roma population in Spain: a population-based national study. Public Health 129 (5), 453–459. 10.1016/j.puhe.2015.01.028 25795016

[B34] Massuda A. Hone T. Leles F. A. G. de Castro M. C. Atun R. (2018). The Brazilian health system at crossroads: progress, crisis and resilience. BMJ Glob. Health 3 (4), e000829. 10.1136/bmjgh-2018-000829 PMC6035510 29997906

[B35] Mokdad A. H. Ballestros K. Echko M. Glenn S. Olsen H. E. Mullany E. (2018). The State of US Health 1990-2016. Jama 319 (14), 1444–1472. 10.1001/jama.2018.0158 29634829 PMC5933332

[B36] Nascimento R. Alvares J. Guerra A. A. J. Gomes I. C. Costa E. A. Leite S. N. (2017a). Availability of essential medicines in primary health care of the Brazilian Unified Health System. Rev. Saude Publica 51 (suppl 2), 10s. 10.11606/S1518-8787.2017051007062 PMC5676352 29160448

[B37] Nascimento R. Alvares J. Guerra A. A. J. Gomes I. C. Silveira M. R. Costa E. A. (2017b). Polypharmacy: a challenge for the primary health care of the Brazilian Unified Health System. Rev. Saude Publica 51 (suppl 2), 19s. 10.11606/S1518-8787.2017051007136 29160460 PMC5676396

[B38] Ofori-Asenso R. (2016). A closer look at the World Health Organization’s prescribing indicators. J. Pharmacol. Pharmacother. 7 (1), 51–54. 10.4103/0976-500X.179352 27127400 PMC4831494

[B39] Oliveira M. A. Luiza V. L. Tavares N. U. L. Mengue S. S. Arrais P. S. D. Farias M. R. (2016). Access to medicines for chronic diseases in Brazil: a multidimensional approach. Rev. Saúde Pública 50 (suppl 2), 6s. 10.1590/s1518-8787.2016050006161 PMC5157920 27982382

[B40] Ozawa S. Shankar R. Leopold C. Orubu S. (2019). Access to medicines through health systems in low- and middle-income countries. Health Policy Plan 34 (Supplement_3), iii1–iii3. 10.1093/heapol/czz119 31816069 PMC6901066

[B41] Paniz V. M. Fassa A. G. Facchini L. A. Bertoldi A. D. Piccini R. X. Tomasi E. (2008). [Access to continuous-use medication among adults and the elderly in South and Northeast Brazil]. Cad. Saude Publica 24 (2), 267–280. 10.1590/s0102-311x2008000200005 18278273

[B42] Price R. H. M. Graham C. Ramalingam S. (2019). Association between viral seasonality and meteorological factors. Sci. Rep. 9 (1), 929. 10.1038/s41598-018-37481-y 30700747 PMC6353886

[B43] Ramos L. R. Tavares N. U. Bertoldi A. D. Farias M. R. Oliveira M. A. Luiza V. L. (2016). Polypharmacy and Polymorbidity in Older Adults in Brazil: a public health challenge. Rev. Saude Publica 50 (suppl 2), 9s. 10.1590/S1518-8787.2016050006145 PMC5157903 27982377

[B44] Santos T. R. Lima D. M. Nakatani A. Y. Pereira L. V. Leal G. S. Amaral R. G. (2013). Medicine use by the elderly in Goiania, Midwestern Brazil. Rev. Saude Publica 47 (1), 94–103. 10.1590/s0034-89102013000100013 23703135

[B45] Shaghaghi A. Asadi M. Allahverdipour H. (2014). Predictors of Self-Medication Behavior: A Systematic Review. Iran J. Public Health 43 (2), 136–146. 26060736 PMC4450680

[B46] Silva M. T. Galvao T. F. (2017). Use of health services among adults living in Manaus metropolitan region, Brazil: population-based survey, 2015. Epidemiol Serv Saude 26, 725–734. 29069169 10.5123/S1679-49742017000400005

[B47] Silva M. T. Nunes B. P. Galvao T. F. (2019). Use of health services by adults in Manaus 2019: Protocol of a population-based survey. Med. (Baltimore) 98 (21), e15769. 10.1097/MD.0000000000015769 PMC6571251 31124966

[B48] Stolk P. Willemen M. J. Leufkens H. G. (2006). Rare essentials: drugs for rare diseases as essential medicines. Bull. World Health Organ. 84 (9), 745–751. 10.2471/blt.06.031518 17128345 PMC2627463

[B49] Stopa S. R. Malta D. C. Monteiro C. N. Szwarcwald C. L. Goldbaum M. Cesar C. L. G. (2017). Use of and access to health services in Brazil 2013 National Health Survey. Rev. Saúde Pública 51 (suppl 1), 3s. 10.1590/s1518-8787.2017051000074 PMC5676359 28591351

[B50] Thiese M. S. (2014). Observational and interventional study design types; an overview. Biochem. Med. (Zagreb) 24 (2), 199–210. 10.11613/bm.2014.022 24969913 PMC4083571

[B51] Trindade J. S. (2018). O fim da Rede Própria do Programa Farmácia Popular do Brasil e o Princípio da Proibição do Retrocesso Social. Cadernos Ibero-Americanos Direito SanitÁrio 7 (2), 61–81. 10.17566/ciads.v7i2.480

[B52] Viacava F. Oliveira R. A. D. Carvalho C. C. Laguardia J. Bellido J. G. (2018). SUS: supply, access to and use of health services over the last 30 years. Cien Saude Colet 23 (6), 1751–1762. 10.1590/1413-81232018236.06022018 29972484

[B53] Vogler S. Osterle A. Mayer S. (2015). Inequalities in medicine use in Central Eastern Europe: an empirical investigation of socioeconomic determinants in eight countries. Int. J. Equity Health 14, 124. 10.1186/s12939-015-0261-0 26541292 PMC4635528

[B54] Weiner S. (2001). “I can’t afford that!”: dilemmas in the care of the uninsured and underinsured. J. Gen. Intern. Med. 16 (6), 412–418. 10.1046/j.1525-1497.2001.016006412.x 11422639 PMC1495228

[B55] WHO (2019). WHO Collaborating Centre for Drug Statistics Methodology (WHOCC). ATC/DDD Index (Oslo: Norwegian Institute of Public Health). Available at: https://www.whocc.no/atc_ddd_index/?code=J&showdescript (Accessed 13 December 2019 2019).

[B56] Wirtz V. J. Kaplan W. A. Kwan G. F. Laing R. O. (2016). Access to medications for cardiovascular diseases in low- and middle-income countries. Circulation 133 (21), 2076–2085. 10.1161/CIRCULATIONAHA.115.008722 27217433 PMC4880457

