# Supplementary material for: Consumption and Lack of Access to Medicines and Associated Factors in the Brazilian Amazon: A Cross-Sectional Study, 2019
Source: Front Pharmacol. 2020 Oct 6;11:586559. doi: 10.3389/fphar.2020.586559 (PMC7573467; doi:10.3389/fphar.2020.586559)
Supplement: Supplementary file 1 [file DataSheet_1.docx]

**SUPPLEMENTARY MATERIAL**

**Supplementary Material 1.** Recruitment procedures of the participants of the study, Manaus, 2019 (N=2,321).

|  | Population ≥18 years old:  2,106,355 in 2,461 census tracts | | | |  |  |  |  |  |
| --- | --- | --- | --- | --- | --- | --- | --- | --- | --- |
|  |  |  |  |  |  |  | | | |
|  |  |  |  |  |  |  |  |  |  |
|  | 5,769 households approached | | | |  |  |  |  |  |
|  |  |  |  |  |  | 2,523 closed or empty households | | | |
|  |  |  |  |  |  |  |  |  |  |
|  | 3,246 households with adult individuals invited to participate in the study | | | |  |  |  |  |  |
|  |  |  |  |  |  | 84 non-eligible individuals and  845 refusals | | | |
|  |  |  |  |  |  |  |  |  |  |
|  | 2,321 participants | | | |  |  |  |  |  |
|  |  |  |  |  |  |  |  |  |  |
