# Supplementary material for: Consumption and Lack of Access to Medicines and Associated Factors in the Brazilian Amazon: A Cross-Sectional Study, 2019
Source: Front Pharmacol. 2020 Oct 6;11:586559. doi: 10.3389/fphar.2020.586559 (PMC7573467; doi:10.3389/fphar.2020.586559)
Supplement: Supplementary file 2 [file DataSheet_2.docx]

**Supplementary Material 2.** Person who recommended or prescribed the medicines used in the 15 days prior to the interview in Manaus, 2019 (N=2,702).

| **Person who recommended or prescribed the medicine** | **N** | **%** |
| --- | --- | --- |
| Physician | 1,839 | 68.1 |
| Own account | 515 | 19.1 |
| Pharmacist or pharmacy clerk | 187 | 6.9 |
| Family member or neighbor | 130 | 4.8 |
| Other | 30 | 1.1 |
